# Trends and perioperative mortality in gastric cancer surgery: a nationwide population‑based cohort study

**DOI:** 10.1007/s13304-023-01632-2

**Published:** 2023-08-24

**Authors:** Roberto Peltrini, Barbara Giordani, Giorgia Duranti, Renato Salvador, Mario Costantini, Francesco Corcione, Umberto Bracale, Giovanni Baglio

**Affiliations:** 1 Department of Public Health, School of Medicine and Surgery, University of Naples Federico II, Via Pansini 5, 80131 Naples, Italy; 2 Research and International Relations Unit, Italian National Agency for Regional Healthcare Services, 00187 Rome, Italy; 3 Department of Surgical, Oncological and Gastroenterological Sciences, University of Padua, 35128 Padua, Italy; 4 Department of Medicine, Surgery and Dentistry, University of Salerno, 84081 Baronissi, SA Italy

**Keywords:** Gastric cancer, Laparoscopy, Gastrectomy, Mortality, Hospital volume

## Abstract

This study aimed to investigate changes and perioperative mortality over a 6-year period within the Italian Hospital Information System among patients with gastric cancer (GC) who underwent gastrectomies and to identify risk factors associated with 90-day mortality. Additionally, nationwide differences between high and low-volume hospitals were evaluated. A nationwide retrospective study was conducted using patient hospital discharge records (HDRs) based on the International Classification of Diseases, 9th Revision, Clinical Modification (ICD-9-CM) classification. The HDRs were linked to the National Tax Registry records using deterministic record linkage. The data were obtained from the Italian National Outcomes Evaluation Programme (PNE). Multivariate logistic regression was used to examine risk factors for 90-day mortality among patients with GC who underwent partial or total gastrectomies over the period from 2018 to 2020 with adjustment for comorbidities. Overall, the number of patients with GC who underwent total or partial gastrectomies steadily decreased in Italy from 5765 in 2015 to 4291 in 2020 (*p* < 0.001). The use of the laparoscopic approach more than doubled from 2015 (10.8%) to 2020 (26.3%), with a concomitant conversion rate from laparoscopy to open surgery decreasing from 7.7 to 5.8%. The 30 and 90-day mortality rates remained stable over time (*p* > 0.05). Low-volume hospitals had higher inpatient, early, and late mortality compared to high-volume hospitals (5.9% vs 3.8%, 6.3% vs 3.8%, and 11.8% vs 7.9%, respectively; *p* < 0.001). Multivariate logistic regression analysis showed that an advanced age (adjusted odds ratio: 3.72; 95% [CI]: 3.15–4.39; *p* < 0.001), an open approach (adjusted-OR: 1.69, 95% CI: 1.43–1.99, *p* < 0.001) and a total gastrectomy (adjusted-OR: 1.44, 95% CI: 1.27–1.64, *p* < 0.001) were independent risk factors for 90-day mortality. Additionally, patients with GC who referred to high-volume hospitals were 26% less likely to die within 90 days after a gastrectomy than those who underwent surgery in low-volume hospitals. During the 6-year period, surgeons implemented a minimally invasive approach to reduce the conversion over time. Centralisation was associated with better outcomes while advanced age, an open approach, and total gastrectomy were identified as risk factors for 90-day mortality.

## Introduction

Gastric cancer (GC) is the fifth most common malignancy and the third leading cause of cancer-related death worldwide [1]. In 2017, more than 1.22 million cases of stomach cancer occurred worldwide, and nearly 865,000 people died of stomach cancer [2]. In Italy, there were approximately 14,700 new GC diagnoses in 2022 and 8500 GC-related deaths in 2021 [3]. GC treatment patterns have evolved constantly. Over the last few decades, efforts have been made to improve patient-related outcomes. A multimodal approach with perioperative chemotherapy was established in the current guidelines, as well as the need for multidisciplinary teams for the modern management of GC [4]. Furthermore, the chance to perform gastrectomy using a minimally invasive approach without compromising survival for locally advanced GC is consolidated not only in Eastern countries [5, 6] but also in Western populations [7–9] when experienced surgeons are available in high-volume centres. The increase in the use of the laparoscopic approach and the trend towards the centralisation of care have inevitably influenced the outcomes of patients with GC in Italy.

The aim of the current study was to provide a comprehensive and real overview of the changing trends and perioperative mortality of patients with GC who underwent gastrectomy within the Italian hospital information system (HIS) over a 6-year period and to investigate the risk factors associated with 90-day mortality. Additionally, we investigated the nationwide impact of hospital volume on GC treatment.

## Methods

### Source and data collection

A nationwide retrospective study was conducted using patient hospital discharge records (HDRs) based on the International Classification of Diseases, 9th Revision, Clinical Modification (ICD-9-CM) classification, provided by more than 1300 public and private Italian hospitals from the National HIS. Hospital discharge data are routinely collected by the Italian Ministry of Health and contain patient demographic information (sex and age), admission and discharge dates, up to six discharge diagnoses (ICD-9-CM), medical procedures or surgical-related characteristics, and status at discharge (alive, dead, or transferred to another hospital). In addition, the National Tax Registry was used to determine vital status or death after hospitalisation. The HDR was linked to the National Tax Registry records using deterministic record linkage.

Data were obtained using the National Outcomes Evaluation Programme (PNE) [10]. All patients discharged from ordinary wards between 2015 and 2020 with a primary diagnosis of gastric cancer [ICD-9-CM code 151.1, 151.2, 151.3, 151.4, 151.5, 151.6, 151.8, or 151.9] who underwent a total or partial gastrectomy procedure [ICD-9-CM codes 43.5–43.9] were enrolled in the study. Partial gastrectomy includes both proximal and distal gastrectomy with esophageal, duodenal, or jejunal anastomosis. Patients with GC who were diagnosed with gastric cardia cancer (Siewert classification esophagogastric junction tumour: type III) were excluded because of different prognoses and management.

Tumour site, type of gastrectomy (partial or total), length of hospital stay (LOS), early mortality (within 30 days of the index hospitalisation), late mortality (within 90 days of the procedure), surgical approach (laparoscopy, open, conversion to open surgery), hospital volume expressed as the number of procedures per year, hospital geographical area, and patient mobility through Italian regions were analysed.

Finally, patients’ comorbidities were gathered over the current and previous 5 years that could affect the outcome, according to the PNE gastric cancer protocol [10].

## Statistical analyses

The first descriptive analysis was performed to show the trends in demographic and clinical variables. Continuous variables were analysed using the Cochran-Armitage trend test, whereas differences among medians were tested using the Kruskal–Wallis test. Hospital facilities were dichotomised into low-volume (≤ 21 procedures/year) and high-volume hospitals (> 21 procedures/year) [11, 12]. Multivariate logistic regression adjusted for baseline comorbidities and geographical area was used to evaluate risk factors of 90-day mortality among patients with GC who underwent gastrectomy over the period from 2018 to 2020.

Patients’ selection and all statistical analyses were performed using SAS Studio 3.81 (Enterprise edition) with a *p* value < 0.05 considered statically significant.

## Results

The baseline characteristics of the patients involved from 1 January 2015 to 31 December 2020 are detailed in Table 1. Overall, the number of patients with GC who underwent total or partial gastrectomies steadily decreased from 5765 in 2015 to 4291 in 2020 (*p* < 0.001).

**Table 1:** Baseline demographic and clinical characteristics of gastric cancer patients undergoing partial or total gastrectomy in Italy (2015–2020)

	2015	2016	2017	2018	2019	2020	*p*-value
Patients (*n*)	5765	5621	5358	5239	4982	4291	< 0.001
Median age (IQR)	74(14)	74(14)	74(14)	74(14)	74(15)	74(14)	0.107
Age—years (%)							< 0.001
< 70	36.8	34.9	35.9	33.9	34.5	33.9	
70–80	42.8	42.7	41.4	41.4	40.4	41.5	
> 80	20.5	22.4	22.7	24.7	25.1	24.7	
Gender (%)							0.602
Male	59.2	57.9	58.8	58.0	57.7	59.0	
Female	40.9	42.1	41.2	42.0	42.3	41.0	
Tumour location (*n*)							< 0.001
Antrum and pylorus (%)	1700 (29.5)	1684 (30.0)	1447 (27.0)	1349 (25.8)	1255 (25.2)	1019 (23.8)	
Fundus, corpus and other locations of the stomach (%)	4065 (70.5)	3937 (70.0)	3911 (73.0)	3890 (74.2)	3727 (74.8)	3272 (76.2)	
Type of surgery (*n*) (%)							< 0.001
Partial gastrectomy (%)	3721 (64.5)	3687 (65.6)	3545 (66.2)	3548 (67.7)	3346 (67.2)	3032 (70.7)	
Total gastrectomy	2044 (35.5)	1934 (34.4)	1813 (33.8)	1691 (32.3)	1636 (32.8)	1259 (29.3)	
Surgical approach (*n*):							< 0.001
Laparoscopy	623 (10.8)	713 (12.7)	887 (16.6)	1045 (20.0)	1153 (23.1)	1127 (26.3)	
Open (%)	5090 (88.3)	4840 (86.1)	4386 (81.9)	4124 (78.7)	3760 (75.5)	3095 (72.1)	
Conversion to open surgery (%)	52 (0.9)	68 (1.2)	85 (1.6)	70 (1.3)	69 (1.4)	69 (1.6)	
Conversion rate (%) to open surgery	7.7	8.7	8.7	6.3	5.6	5.8	< 0.001
Mobility outside region of origin for treatment (%)	496 (8.6)	508 (9.0)	492 (9.2)	540 (10.3)	467 (9.4)	374 (8.7)	0.294

Approximately, 59% of the patients who met the selection criteria were male and a positive trend in patients who underwent surgery for GC for > 80 years (*p* < 0.001) was observed in this timeframe.

**Fig. 1:**
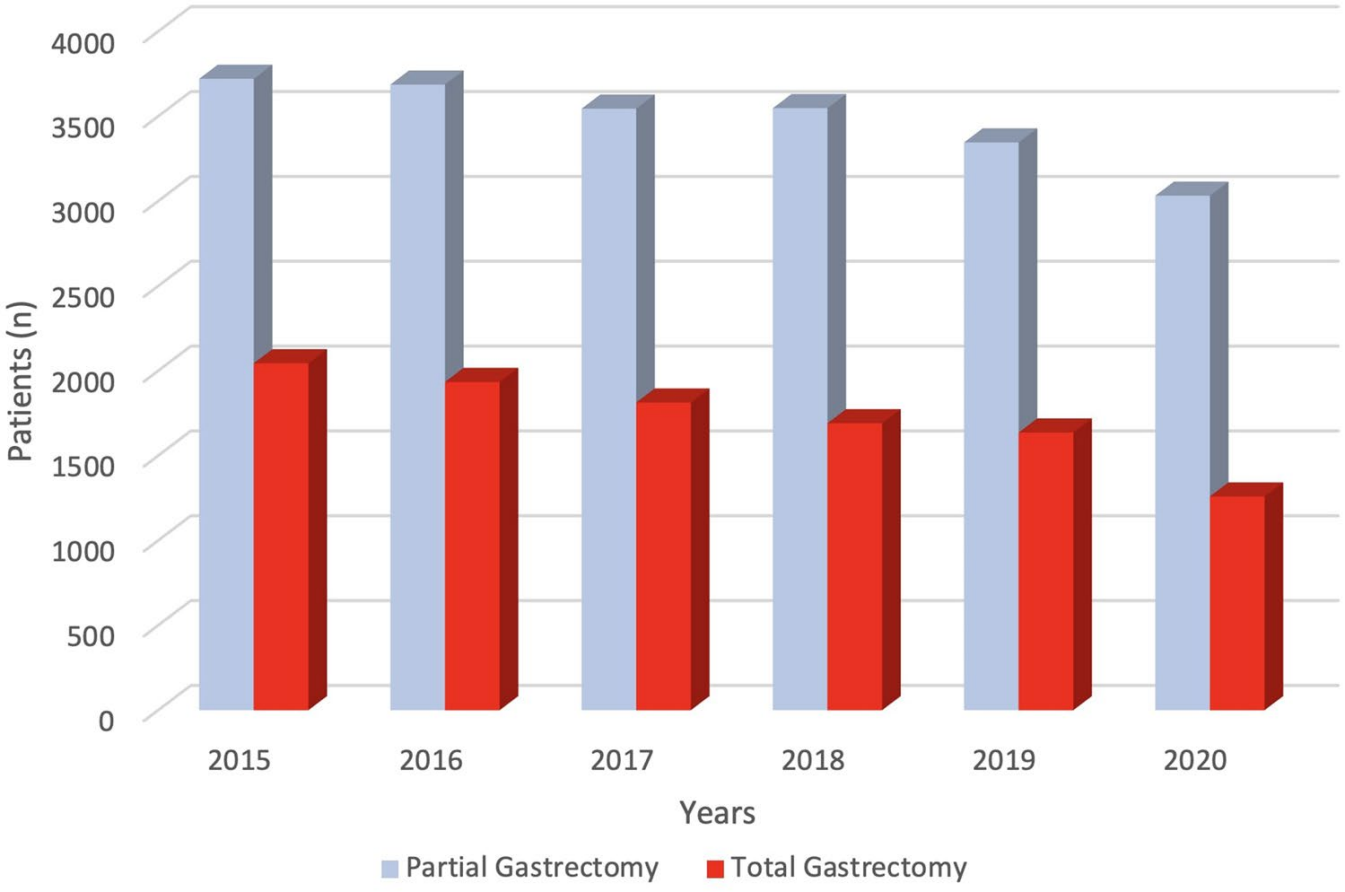
Distribution of patients by surgical procedures (partial and total gastrectomy) in the study period (*p* < 0.001)

**Fig. 2:**
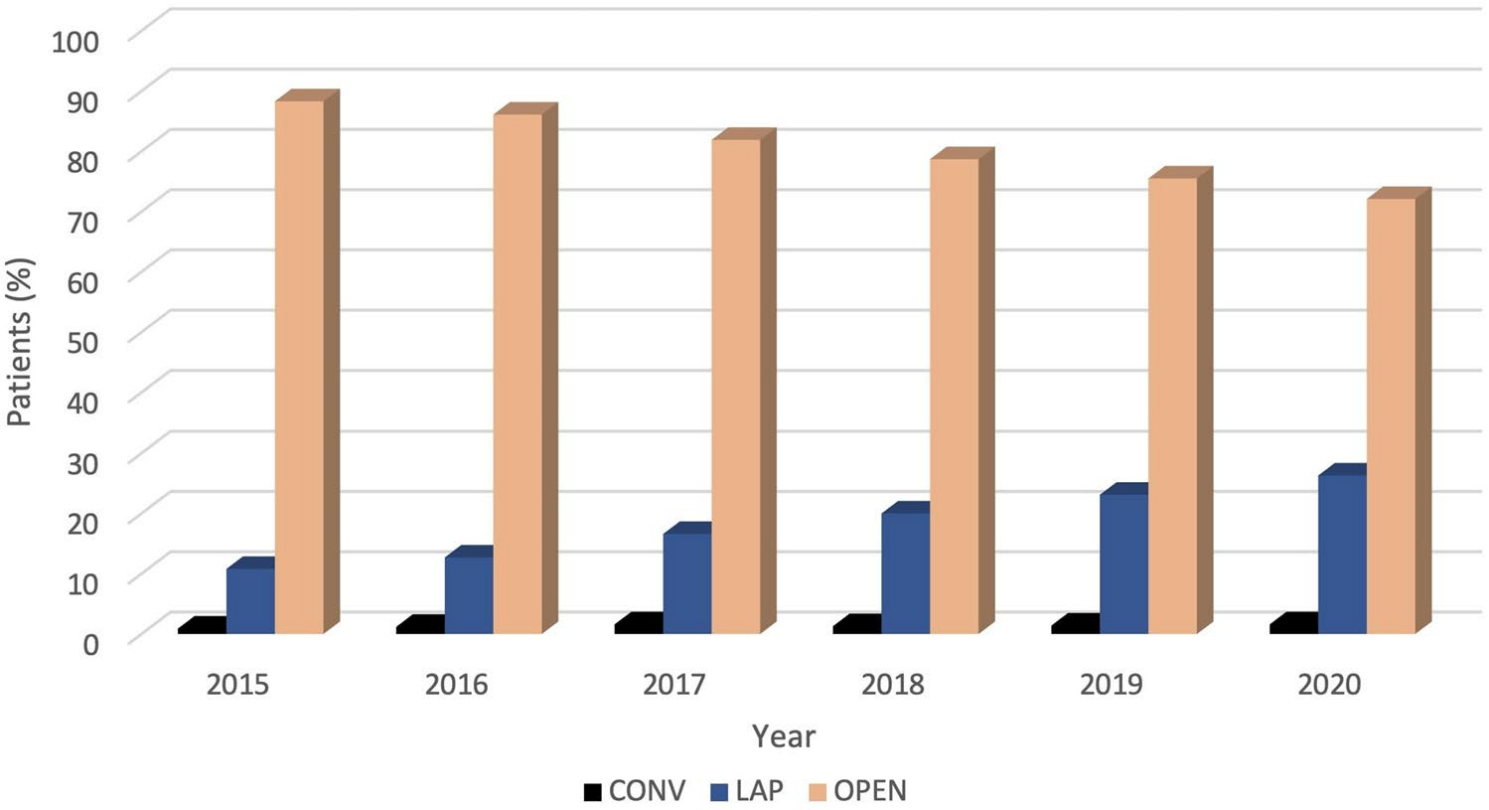
Distribution of patients by surgical approaches (open, laparoscopic and conversion to open surgery) in the study period (*p* < 0.001)

The number of total gastrectomies decreased significantly over time from 35.5% in 2015 to 29.3% in 2020 (*p* < 0.001), and, at the same time, the proportions of patients who underwent partial gastrectomies increased from 64.5 to 70.7% (Fig. 1). Although a steady decline in the number of patients was observed from 2015 to 2020, the use of laparoscopy (Fig. 2) more than doubled from 2015 (10.8%) to 2020 (26.3%), with a concomitant conversion rate from laparoscopic to open surgery decreasing from 7.7 to 5.8% (*p* < 0.001). No evidence was found of changes in patient mobility between the regions of the country over the analysis time.

Table 2 shows trends in postoperative outcomes. Early and late mortality remained stable over time and the median hospital stay (from the day of surgery) significantly decreased by 1 day (*p* < 0.001).

**Table 2:** Postoperative outcomes of GC patients who underwent partial or total gastrectomy in Italy (2015–2020)

	2015	2016	2017	2018	2019	2020	*p*-value
Patients (*n*)	5765	5621	5358	5239	4982	4291	< 0.001
Length of hospital stay—days (IQR)	11 (6)	11 (6)	11 (7)	10 (6)	10 (6)	10 (6)	< 0.001
In-hospital mortality (%)	331 (5.7)	308 (5.5)	247 (4.6)	256 (4.9)	246 (4.9)	218 (5.1)	0.049
30-day mortality (%) *	343 (6.0)	308 (5.5)	264 (4.9)	248 (4.7)	262 (5.3)	243 (5.7)	0.256
90-day mortality (%) *	639 (11.1)	605 (10.8)	529 (9.9)	502 (9.6)	503 (10.1)	460 (10.7)	0.162

*Including deaths during recovery

The comparative analysis between low and high-volume centres from 2018 to 2020 (Table 3) showed that patients with GC were significantly older in hospitals with ≤ 21 procedures/year (mean age 73.2 vs 70.9 and median age 75 vs 73 years); this was also confirmed by the prevalence of patients > 80 years (27.5% vs 21.4%, *p* < 0.001). There was strong evidence that high-volume hospitals performed more total gastrectomies with a higher rate of laparoscopic approaches than low-volume centres (*p* < 0.001). In addition, the median hospital stay after surgery was 2 days shorter in high-volume hospitals (*p* < 0.001).

**Table 3:** Distribution of GC patients operated on by hospital volume in Italy (2018–2020)

	Hospitals with > 21 gastrectomy/year *N* patients = 6304	Hospitals with ≤ 21 gastrectomy/year *N* patients = 8208	*p*-value
Median age (IQR)	73 (16)	75 (14)	< 0.001
Age—years (%)			< 0.001
< 70	38.7	30.6	
70–80	39.9	42.0	
> 80	21.4	27.5	
Gender (%)			0.475
Male	58.5	57.9	
Female	41.5	42.1	
Tumour location (%)			0.191
Antrum and pylorus	24.4	25.4	
Fundus, corpus and other locations of the stomach	75.6	74.6	
Type of surgery (%)			< 0.001
Partial gastrectomy	64.9	71.1	
Total gastrectomy	35.1	28.9	
Surgical approach (%)			< 0.001
Laparoscopy	26.1	20.5	
Open	72.6	78.0	
Conversion to open surgery	1.3	1.5	
Lenght of hospital stay—days (IQR)	9 (6)	11 (7)	< 0.001
90-day mortality (%)	7.9	11.8	< 0.001
30-day mortality (%)	3.8	6.3	< 0.001
In-hospital mortality (%)	3.8	5.9	< 0.001
Mobility outside region of origin for treatment (%)	14.9	5.4	< 0.001

Patients with GC who underwent surgery in low-volume hospitals had higher inpatient, early, and late mortality (5.9% vs 3.8%, 6.3% vs 3.8%, and 11.8% vs 7.9%, respectively; *p* < 0.001). Furthermore, approximately 15% of patients referred to high-volume hospitals for treatment moved out of the region.

Multivariate logistic regression analysis (Table 4) showed that older patients (adjusted odds ratio: 3.72; 95% [CI]: 3.15–4.39; *p* < 0.001) are almost four times more likely to die within 90 days than younger patients, while females were about 13% less likely to die than males. The open approach and total gastrectomies were strongly associated with 90-day mortality (adjusted-OR: 1.69, 95% CI: 1.43–1.99, *p* < 0.001; adjusted OR: 1.44, 95% CI: 1.27–1.64, *p* < 0.001, respectively). In addition, patients with GC who were referred to high-volume hospitals were 26% less likely to die within 90 days of gastrectomy than those who underwent surgery in low-volume hospitals (*p* < 0.001).

**Table 4:** Multivariate logistic regression analysis for the evaluation of risk factors of 90-day mortality among GC patients undergoing partial or total gastrectomy (*N* patients = 14,512) in Italy (2018–2020), adjusted for geographical area and comorbidities (diabetes, obesity, lipid metabolism disorders, anaemia, coagulation defects, other haematological diseases, arterial hypertension, ischemic heart disease, previous coronary revascularisation, heart failure, other heart diseases/operations, arrhythmias, brain circulatory disorders, chronic obstructive pulmonary disease and chronic respiratory failure, chronic kidney failure, moderate/severe liver disease, chronic inflammatory bowel disease, pancreatic disease, hemiplegia and other paralysis, dementia including Alzheimer and Parkinson disease, malnutrition, dehydration, disorders of acid–base balance, cachexia, previous surgery of gastrectomy)

	*N*	OR*	95% CI		*p*-value
Age (years)					
< 70	4948	1			
70–80	5963	1.876	1.599	2.201	< .0001
> 80	3601	3.718	3.152	4.385	< .0001
Gender					
Male	8446	1			
Female	6066	0.874	0.777	0.983	0.025
Tumour location					
Fundus, corpus and other locations of the stomach	10,889	1			
Antrum and pylorus	3623	1.078	0.941	1.235	0.280
Surgical approach					
Laparoscopy	3325	1			
Conversion to open surgery`	208	1.875	1.165	3.018	0.010
Open	10,979	1.691	1.437	1.990	< .0001
Type of surgery					
Partial gastrectomy	9926	1			
Total gastrectomy	4586	1.441	1.270	1.636	< .0001
Mobility outside region of origin for treatment					
No	13,131	1			
Yes	1381	0.859	0.685	1.076	0.186
Hospital volume					
≤ 21 gastrectomy/year	8208	1			
> 21 gastrectomy/year	6304	0.738	0.654	0.832	< .0001

## Discussion

The present study provides an overview of the trends and achievements during 6 years of GC surgery in Italy using the Italian National Healthcare Outcomes Program (PNE 2022).

Laparoscopic gastrectomy significantly increased from 10.8 in 2015 to 26.3% in 2020, and a concomitant reduction was observed for the open approach. In addition, conversion to open surgery decreased over time as indirect evidence of proficiency. A similar trend emerged from data analysis of the National Cancer Database (NCDB) between 2010 and 2015 [13]. In the USA, the use of minimally invasive approaches for gastrectomies has increased annually with improved oncologic outcomes, while the use of open operations has declined each year. In contrast, the laparoscopic gastrectomy rate reached 40.8% in 2013 in the East, based on the Japanese Gastric Cancer Association Registry data [14]. This discrepancy with the percentage found in the present study was probably due to the paucity of evidence on long-term outcomes following laparoscopic resection at the time and the limited application of Eastern data on Western populations (BMI, comorbidities, and different biological cancer behaviours) [15].

The 30- and 90-day mortality rates after GC surgery remained relatively stable, ranging from 4.7–6.0% to 9.6–11.1%, respectively, over the study period. These findings are consistent with those of previous studies. Data from the French National Health Service database, including 11,196 patients with gastric (70%) and oesophageal cancer (30%), showed 30- and 90-day postoperative mortality rates of 5% and 9%, respectively [16]. Similarly, in a Dutch population of 4,837 patients with GC, the 30-day mortality rate was approximately 6% [17]. However, centralisation played a pivotal role in both studies, as it was associated with improved oncologic outcomes. Comparing high- and low-volume hospitals, in our analysis, we observed not only lower readmission and mortality rates but also higher use of a minimally invasive approach and total gastrectomy in hospitals with > 21 procedures/years. A significant and strong association between hospital volume and survival benefits for patients with gastric cancer surgery has been widely recognised [12, 18, 19]. In the current study, the hospital high volume was emphasised in the multivariate regression analysis as an independent protective factor for 90-day mortality. However, particular attention should be paid to the relationship between mortality rate and surgeon volume. Regarding gastric cancer surgery, patients treated by experienced surgeons have a better prognosis, with improved survival and lower locoregional recurrence and anastomotic leakage rates [20–22].

The open approach and total gastrectomies were strongly associated with 90-day mortality. These findings are consistent with those of a recent Swedish population-based cohort study, including 622 patients with GC. Compared with open surgery, the laparoscopic approach was associated with significantly lower 30- and 90-day mortality rates [23]. Similarly, the advantages of laparoscopy in terms of 90-day mortality were confirmed in a French cohort study of 10,343 patients who underwent both distal and total gastrectomy [24]. However, conflicting results remain in the literature [25], and several factors that could affect the prognosis should be considered, such as neoadjuvant treatment and lymph node dissection [26, 27]. Furthermore, it is reasonable to assume that larger or more advanced tumours were treated with open gastrectomy in many hospitals, partially limiting the consistency of our finding.

The main strengths of this study are the large sample size and real-world data from a Western population-based perspective. However, this study had some limitations. Because the registration of baseline characteristics in the HIS is limited, relevant information regarding the stage of the disease, neoadjuvant therapy, lymph node dissection, and histopathological details are not available. Furthermore, the quality of the data entered from each hospital may have been prone to bias (i.e. underreporting, miscoding), and the impact of Sars-Cov-2 on elective surgical activity in 2020 was not considered in the analysis. Another limitation is the cutoff for surgical procedures to define high and low-volume centres, which have been defined differently over the years and in different countries.

## Conclusion

The awareness and a comprehensive critical assessment of GC “state-of-art” in a large-scale population may help to better understand the quality of care and improve the future decision-making process with potential implications in the clinical practice. During the 6-year period, we recorded the unmodified early and late mortality rates after GC surgery. Surgeons have implemented a minimally invasive approach to reduce the conversion rate over time. Centralisation was associated with better outcomes and advanced age, open approach, and total gastrectomy were identified as risk factors for 90-day mortality.

## Data Availability

The data presented in this study are available from the authors upon reasoned request.

## References

[1] Bray F, Ferlay J, Soerjomataram I, Siegel RL, Torre LA, Jemal A (2018). Global cancer statistics 2018: GLOBOCAN estimates of incidence and mortality worldwide for 36 cancers in 185 countries. CA Cancer J Clin.

[2] GBD 2017 Stomach Cancer Collaborators (2020). The global, regional, and national burden of stomach cancer in 195 countries, 1990–2017: a systematic analysis for the Global Burden of Disease study 2017. Lancet Gastroenterol Hepatol..

[3] I numeri del cancro in Italia 2022 – Rapporto AIOM-AIRTUM. https://www.aiom.it/wp-content/uploads/2022/12/2022_AIOM_NDC-web.pdf Accessed 19 May 2023.

[4] Lordick F, Carneiro F, Cascinu S (2022). Gastric cancer: ESMO clinical practice guideline for diagnosis, treatment and follow-up. Ann Oncol.

[5] Yu J, Huang C, Sun Y (2019). Effect of laparoscopic vs open distal gastrectomy on 3-year disease-free survival in patients with locally advanced gastric cancer: the CLASS-01 randomized clinical trial. JAMA.

[6] Hyung WJ, Yang HK, Park YK (2020). Long-Term outcomes of laparoscopic distal gastrectomy for locally advanced gastric cancer: the KLASS-02-RCT randomized clinical trial. J Clin Oncol.

[7] van der Wielen N, Straatman J, Daams F (2021). Open versus minimally invasive total gastrectomy after neoadjuvant chemotherapy: results of a European randomized trial. Gastric Cancer.

[8] Greenleaf EK, Sun SX, Hollenbeak CS, Wong J (2017). Minimally invasive surgery for gastric cancer: the American experience. Gastric Cancer.

[9] Bracale U, Merola G, Pignata G (2022). Laparoscopic gastrectomy for stage II and III advanced gastric cancer: long-term follow-up data from a Western multicenter retrospective study. Surg Endosc.

[10] AGENAS. The Italian National Outcomes Evaluation Programme 2022 Edition. Available online: https://pne.agenas.it/ (accessed on 24 July 2023).

[11] Birkmeyer JD, Siewers AE, Finlayson EV, Stukel TA, Lucas FL, Batista I, Welch HG, Wennberg DE (2002). Hospital volume and surgical mortality in the United States. N Engl J Med.

[12] Claassen YHM, van Amelsfoort RM, Hartgrink HH (2019). Effect of hospital volume with respect to performing gastric cancer resection on recurrence and survival: results from the CRITICS trial. Ann Surg.

[13] Hendriksen BS, Brooks AJ, Hollenbeak CS, Taylor MD, Reed MF, Soybel DI (2020). The impact of minimally invasive gastrectomy on survival in the USA. J Gastrointest Surg.

[14] Kakeji Y, Ishikawa T, Suzuki S (2022). A retrospective 5-year survival analysis of surgically resected gastric cancer cases from the Japanese Gastric Cancer Association nationwide registry (2001–2013). Gastric Cancer.

[15] De Manzoni G, Marrelli D, Baiocchi GL (2017). The Italian research group for gastric cancer (GIRCG) guidelines for gastric cancer staging and treatment: 2015. Gastric Cancer.

[16] Pasquer A, Renaud F, Hec F (2016). Is centralization needed for esophageal and gastric cancer patients with low operative risk?: A Nationwide Study. Ann Surg.

[17] Busweiler LAD, Dikken JL, Henneman D (2017). The influence of a composite hospital volume on outcomes for gastric cancer surgery: a Dutch population-based study. J Surg Oncol.

[18] Ji J, Shi L, Ying X, Lu X, Shan F (2022). Associations of annual hospital and surgeon volume with patient outcomes after gastrectomy: a systematic review and meta-analysis. Ann Surg Oncol.

[19] Diers J, Baum P, Wagner JC, Matthes H, Pietryga S, Baumann N, Uttinger K, Germer CT, Wiegering A (2021). Hospital volume following major surgery for gastric cancer determines in-hospital mortality rate and failure to rescue: a nation-wide study based on German billing data (2009–2017). Gastric Cancer.

[20] Liang Y, Wu L, Wang X, Ding X, Liang H (2015). The positive impact of surgeon specialization on survival for gastric cancer patients after surgery with curative intent. Gastric Cancer.

[21] Iwatsuki M, Yamamoto H, Miyata H, Kakeji Y, Yoshida K, Konno H, Seto Y, Baba H (2019). Effect of hospital and surgeon volume on postoperative outcomes after distal gastrectomy for gastric cancer based on data from 145,523 Japanese patients collected from a nationwide web-based data entry system. Gastric Cancer.

[22] Fischer C, Lingsma H, Klazinga N, Hardwick R, Cromwell D, Steyerberg E, Groene O (2017). Volume-outcome revisited: the effect of hospital and surgeon volumes on multiple outcome measures in oesophago-gastric cancer surgery. PLoS ONE.

[23] Tsekrekos A, Vossen LE, Lundell L, Jeremiasen M, Johnsson E, Hedberg J, Edholm D, Klevebro F, Nilsson M, Rouvelas I (2023). Improved survival after laparoscopic compared to open gastrectomy for advanced gastric cancer: a Swedish population-based cohort study. Gastric Cancer.

[24] Challine A, Voron T, Dousset B, Creavin B, Katsahian S, Parc Y, Lazzati A, Lefèvre JH (2021). Postoperative outcomes after laparoscopic or open gastrectomy. A National Cohort Study of 10,343 patients. Eur J Surg Oncol.

[25] Gambhir S, Inaba CS, Whealon M, Sujatha-Bhaskar S, Pejcinovska M, Nguyen NT (2021). Short- and long-term survival after laparoscopic versus open total gastrectomy for gastric adenocarcinoma: a National database study. Surg Endosc.

[26] Bracale U, Corcione F, Pignata G (2021). Impact of neoadjuvant therapy followed by laparoscopic radical gastrectomy with D2 lymph node dissection in Western population: A multi-institutional propensity score-matched study. J Surg Oncol.

[27] Bracale U, Pignata G, Lirici MM (2012). Laparoscopic gastrectomies for cancer: The ACOI-IHTSC national guidelines. Minim Invasive Ther Allied Technol.

